# Effectiveness of creating digital twins with different digital dentition models and cone-beam computed tomography

**DOI:** 10.1038/s41598-023-37774-x

**Published:** 2023-06-30

**Authors:** Joo-Hee Lee, Hye-Lim Lee, In-Young Park, Sung-Woon On, Soo-Hwan Byun, Byoung-Eun Yang

**Affiliations:** 1 Division of Pediatric Dentistry, Hallym University Sacred Heart Hospital, Anyang, 14066 Korea; 2 Division of Oral and Maxillofacial Surgery, Hallym University Sacred Heart Hospital, Anyang, 14066 Korea; 3 Department of Artificial Intelligence and Robotics in Dentistry, Graduate School of Clinical Dentistry, Hallym University, Chuncheon, 24252 Korea; 4 Institute of Clinical Dentistry, Hallym University, Chuncheon, 24252 Korea

**Keywords:** Medical research, Anatomy

## Abstract

Distortion of dentition may occur in cone-beam computed tomography (CBCT) scans due to artifacts, and further imaging is frequently required to produce digital twins. The use of a plaster model is common; however, it has certain drawbacks. This study aimed to assess the feasibility of different digital dentition models over that of plaster casts. Plaster models, alginate impressions, intraoral scan (IOS) images, and CBCT images of 20 patients were obtained. The desktop model scanner was used to scan the alginate impression twice, five minutes and two hours after impression-making. Using an IOS, the full arch was scanned in segments using CS 3600 and simultaneously with i700 wireless. The digital twins obtained from the alginate impression and IOS were superimposed with those obtained from the plaster cast. The differences and distances at each reference point were measured. Scans of alginate impressions after two hours showed the greatest discrepancies, but these were all less than the CBCT voxel size of 0.39 mm. Alginate impression scans and IOS are suitable supplements to CBCT compared to the plaster model. Accuracy can be improved by scanning the alginate impression within five minutes or by intraoral scanning of the entire arch with segmentation.

## Introduction

Recently, dentistry has seen an increase in the use of three-dimensional (3D) digital imaging to develop virtual treatment plans^1–3^. The term "digital twin" refers to data that is recreated in a virtual setting to mimic reality^4,5^. It can help develop and select the most efficacious treatment options for each patient and predict the outcomes. Cone-beam computed tomography (CBCT) is the primary tool used to create digital twins. As virtual surgical planning depends on precise imaging of the dental occlusal surface to determine the occlusion, gathering accurate dental imaging data is crucial^6^. However, the following two factors limit the precision of CBCT scans: 1) streak artifacts are caused by objects such as orthodontic appliances, restorations, implants, and enamel and 2) distortion of images produced by the X-ray beam^7–10^. The occlusal surface of the teeth appears distorted as the images of the teeth become larger. Therefore, CBCT should be combined with supplementary dentition imaging to improve precision.

A technique frequently employed in clinical practice involves superimposing a dentition image produced by a model scan of a plaster model on a CBCT image to create a digital twin of the patient^1,11–13^. Plaster models take up storage space, are prone to loss and damage, are challenging to retrieve, and cannot be easily transferred. Digital models have replaced plaster casts because of recent advancements in digital technology to address the drawbacks of plaster models. Direct scanning of alginate impressions using a desktop scanner was introduced to eliminate the need for plaster pouring. However, alginate materials undergo syneresis and imbibition over time when exposed to the environment. Jiang et al. reported no statistically significant difference in the dimensions of the scan when the alginate impression was scanned at one hour of impression-making, but a significant difference was observed at two hours^14^. They also reported that extended-pour alginate material did not show a statistically significant difference from other traditional alginates.

An intraoral scanner (IOS) requires neither physical impression nor plaster pouring. Additionally, several patients reported being more comfortable during intraoral scanning than during traditional impression-making^15,16^. Lee et al. reported that using IOS satisfactorily complements CBCT compared to the plaster model^17^. Partial arch scan (scanning the arch in three parts and merging the partial scans into a full arch scan) with CS 3600 (Carestream Dental, Atlanta, USA) has shown the best results, followed by the use of i700 (Medit, Seoul, Korea) with three methods (complete arch scan, partial arch scan, and “smart stitch” function) and full-arch scan with CS 3600.

The purpose of this study was to determine whether virtual surgical planning through direct scanning of alginate impressions and IOS is an effective alternative to traditional plaster casts.

## Methods

Twenty patients (9 boys, 11 girls; age range: 12–18 years) who visited the Department of Dentistry at Hallym University Sacred Heart Hospital were included based on the following criteria: complete eruption of the first molar, no cleft palate or craniofacial syndromes, and no metal orthodontic devices or restorations intraorally. The sample size was calculated using G*power (ver. 3.0.10, Franz Faul. Universitat, Kiel, Germany), with a significance level of a = 0.05, 95% power, and an effect size of 0.80. This study was approved by the Institutional Review Board (IRB No. 2020-07-005-001) of Hallym University Sacred Heart Hospital. Methods in this study were performed in accordance with the Declaration of Helsinki. Because the study was retrospective and used anonymized clinical data of participants, the need for informed consent was waived by the Institutional Review Board.

The patients underwent alginate impression-making, intraoral scanning, and CBCT within a two-week period. A retrospective analysis was performed using the maxillary portion of each patient’s plaster model, alginate impressions, IOS images, and CBCT images. The surface of the plaster model (Rhombstone white, Ryoka Dental, Mie-Ken, Japan) and alginate impression (Cavex Holland BV, Haarlem, the Netherlands) were scanned with a desktop model scanner, Freedom UHD (Dof, Inc., Seoul, Korea), to acquire standard tessellation language (STL) format digital images. The IOSs used were CS 3600 (Carestream Dental, Atlanta, USA) and i700 wireless (Medit, Seoul, Korea). One clinician (JH Lee) performed intraoral scanning in accordance with the manufacturer's instructions to obtain STL data. CBCT was performed using Alphard 3030 (Asahi, Inc., Kyoto, Japan), with the Frankfort plane parallel to the horizontal plane, field of view 200 mm × 200 mm, voxel size of 0.39 mm, and exposure conditions of 80 kVp, 5 mA, and 17 s. CBCT images were converted to the Digital Imaging and Communications in Medicine (DICOM) format with 3D reconstruction.

The alginate impression was scanned with the desktop model scanner once within five minutes and then again after two hours. The excess material beyond the impression tray was cut without affecting the cervical region of teeth in the impression. Any contaminants and saliva were rinsed away under flowing water. Water accumulating on the surface of the impression was blown away with gentle air. Each impression was placed in a room without a sealing device to replicate a typical clinical setting, with temperatures between 16 and 21 °C and relative humidity (RH) between 45 and 55%. The full arch was scanned in sections using CS 3600 (prosthetic mode) and simultaneously with i700 wireless. According to Lee et al., a segmented scan performs better than a full-arch scan with CS 3600, whereas comparable results were obtained with i700 regardless of the scan method (full-arch scan, segmented scan, or “smart stitch” feature, which allows common parts between various scan pieces to be joined automatically)^17^. The distal half of the right canine to the distal surface of the rearmost right tooth, the mesial half of the right first premolar to the mesial half of the left first premolar, and the distal half of the left canine to the distal surface of the rearmost left tooth were used as landmarks for the segmented scan. Each segmented image was then semi-automatically merged based on the overlapping scan images in Geomagic Freeform Plus (3D Systems, North Carolina, USA) to create a full-arch dental scan (STL format).

**Figure 1:**
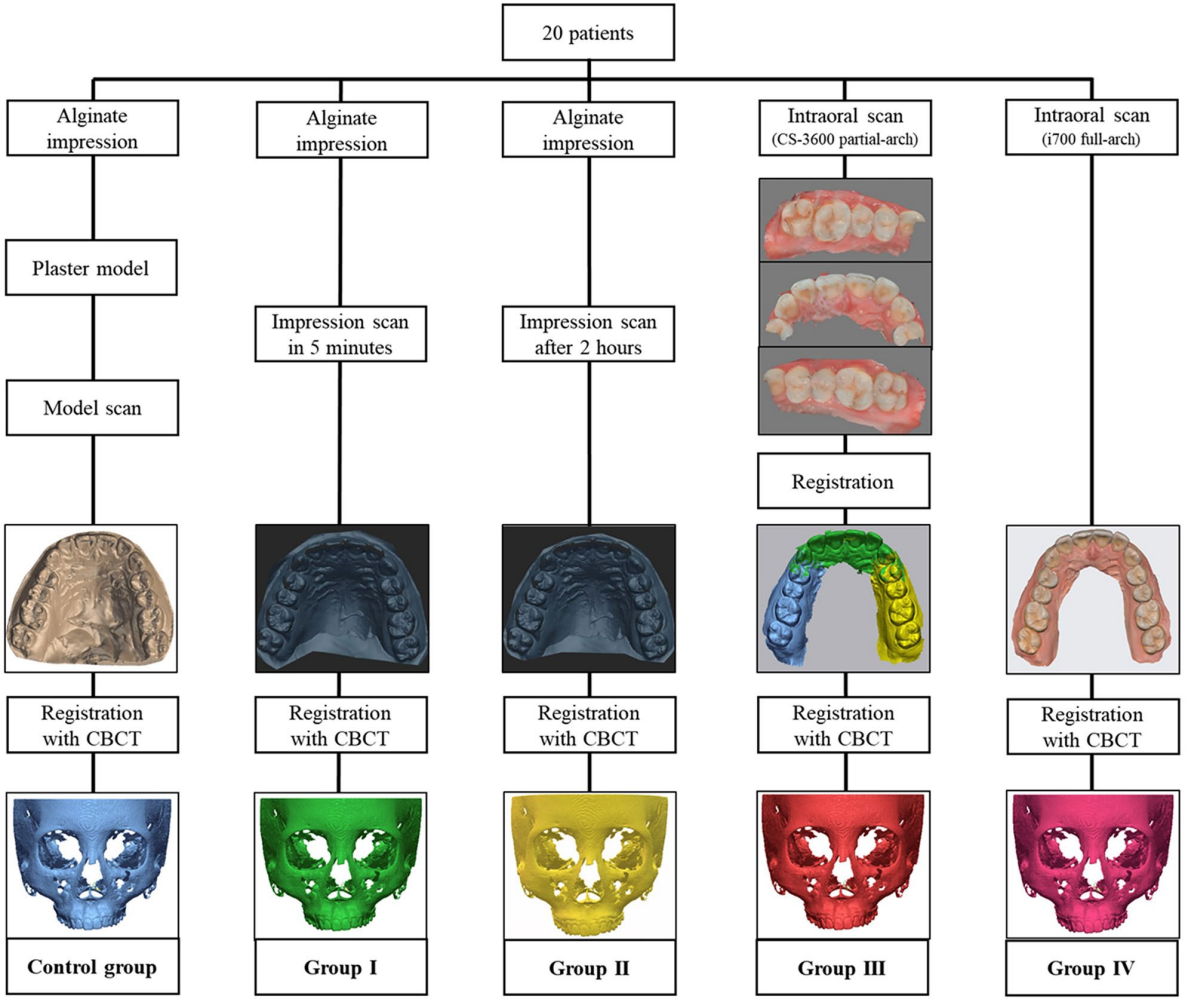
Flow diagram of preparing digital twins in each group. Alginate impressions and intraoral scans were performed during clinical treatment and subsequent procedures were performed separately with the records.

**Figure 2:**
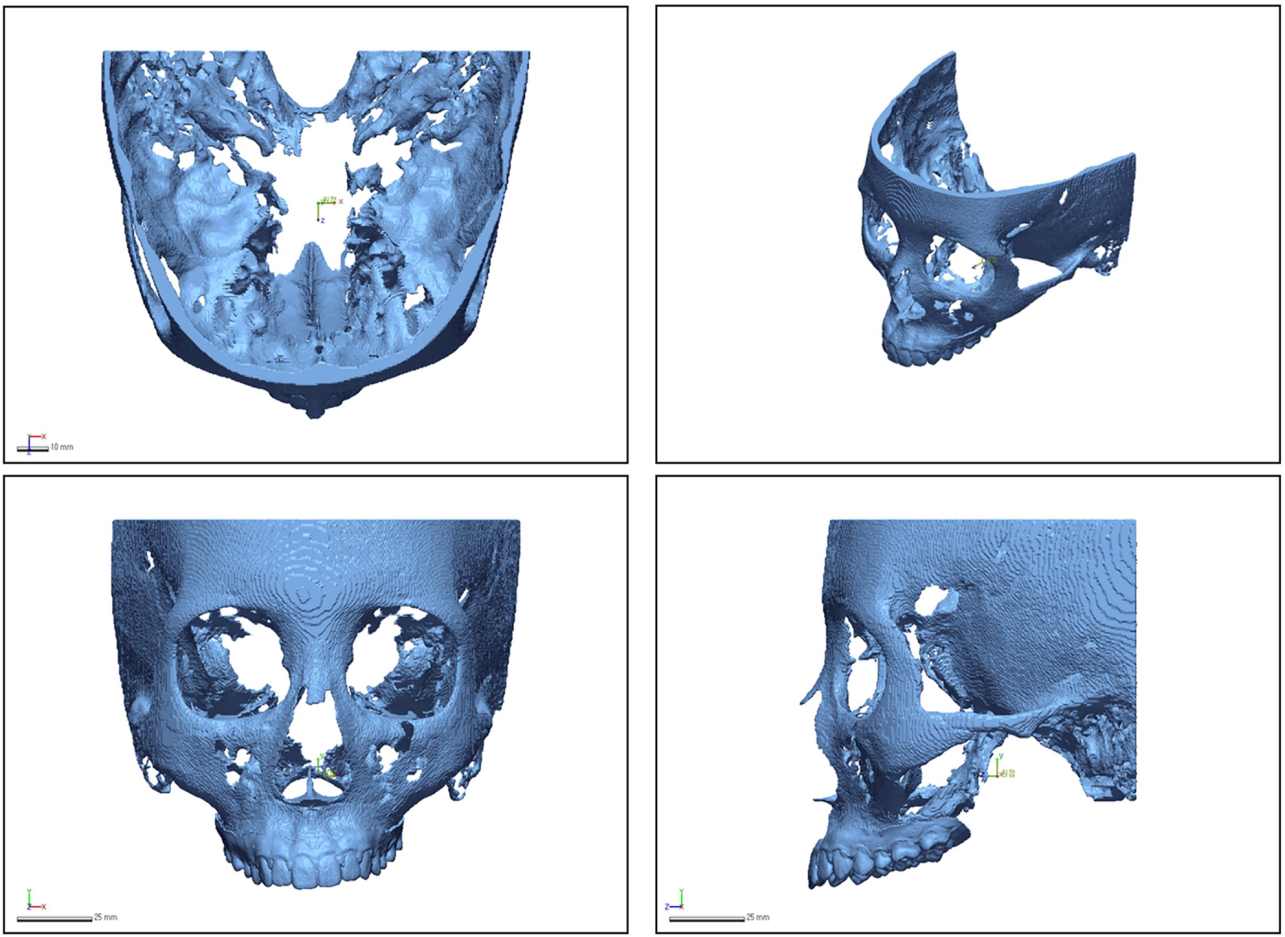
Five final digital twins per patient were generated.

Consequently, five dentition images were created for each patient. The CBCT images (DICOM format) and each dentition scan image (STL format) were transferred to the R2GATE™ program (MegaGen Implant Co., Ltd.). Semi-automatic merging was performed based on the midpoint of the incisal edge of the left and right maxillary central incisors and the mesiobuccal cusp of the left and right maxillary first molars. Five final digital twins per patient were generated (Figs. 1 and 2).

1. *Control group* Dentition image produced via a plaster cast model scan + CBCT scan.
2. *Group I* Dentition image produced via an impression scan of alginate within five minutes of impression-making + CBCT scan.
3. *Group II* Dentition image produced via an impression scan of alginate after two hours of impression-making + CBCT scan.
4. *Group III* Dentition image produced after segmentation scan with CS 3600 + CBCT scan.
5. *Group IV* Dentition image produced by scanning the full arch with an i700 wireless scanner + CBCT scan.

Six reference points (the cusp of the bilateral canines, the most apical point of the gingival margin of the bilateral canines, and the mesiobuccal cusp of the bilateral first molars) were set for each digital twin. The three-dimensional data of each reference point were expressed as x, y, and z coordinate values and entered into a program (Geomagic Freeform Plus). The difference between the coordinate values and the distance between the reference points was calculated by superimposing the digital models of the control and other groups. One-way ANOVA and Tukey’s post-hoc test were used for statistical comparisons. The Statistical Package for Social Sciences (SPSS, version 25.0, IBM) was used to conduct statistical analyses.

## Results

The calculated differences in coordinate values and distances at each reference point are presented in this section. Except for the y-value of the mesiobuccal cusp of the right first molar, there were significant differences between the groups at all reference points. A post-hoc analysis was performed for further evaluation.

The greatest differences in the position of the reference points were observed on the impression scans of alginate impression taken after two hours (Group II). Group II showed more than 0.2 mm at all reference points, whereas the largest distance in the other groups was 0.053 mm. The least difference was observed for dentition images obtained by an impression scan of alginate impression within five minutes of impression-making (Group I) and after the segmentation scan with CS 3600 (Group III). The mean values of the differences were highest in Group II and lowest in Groups I and III (Table 1). Information on coordinate value differences is provided in the supplementary material (Tables S1–S6).

**Table 1:** Statistical analysis of the distance (mm) at the reference points between groups I, II, III, IV, and the control group.

		#13 gm	#13 cusp	#16 mbc	#23 gm	#23 cusp	#26 mbc
I	Average	0.024	0.029	0.010	− 0.017	0.010	0.000
	SD	0.036	0.028	0.017	0.027	0.021	0.012
II	Average	0.217	0.207	0.216	0.208	0.208	0.202
	SD	0.035	0.055	0.018	0.037	0.022	0.011
III	Average	− 0.003	0.002	− 0.011	0.016	− 0.004	− 0.009
	SD	0.026	0.024	0.016	0.044	0.017	0.010
IV	Average	0.018	− 0.042	− 0.045	− 0.008	− 0.053	− 0.042
	SD	0.056	0.021	0.018	0.028	0.060	0.015
F		136.503	196.141	899.840	187.738	210.284	1585.982
p (1)		0.000*	0.000*	0.000*	0.000*	0.000*	0.000*
T (2)		II > I, IV, C, III	II > I, III III > C > IV	II > I, CC > III > IV	II > III, C, IV C, IV > I	II > IV > C, III, I	II > I, C, III > IV

The Fédération Dentaire Internationale two-digit notation system is used to identify teeth.

gm, lowest point of the gingival margin; mbc, mesiobuccal cusp; SD, standard deviation.

(1) Statistical significance was tested using one-way ANOVA among groups (*p < 0.05).

(2) Adjustment for multiple comparisons: Tukey’s test.

## Discussion

The craniomaxillofacial region of a patient can be visualized in 3D using CBCT. To overcome the limitations of CBCT, additional dental images using plaster casts, alginate impressions, and IOSs have been merged with CBCT images to create digital twins. Creating a digital twin patient by superimposing the dental image using a plaster model scan with a CBCT image has been frequently performed in clinical practice. Reproducing reality with a digital twin has great potential for improving clinical treatments^5^. A multidisciplinary team can organize the surgery, and simulation training can be performed prior to the procedure using a digital twin^18^. Digital twins can also be used as a reference during surgery to confirm the anatomy and prevent unintentional structural damage. Maxillofacial surgery can be planned using virtual simulations, and dental wafers and implant surgical guides can be created using computer-aided design and manufacturing techniques^19–22^. When used for orthognathic surgery, it accurately depicts the patient's real dentition in a clinically acceptable way and clearly shows the intended surgical outcome^20,23–26^.

The four digital twin groups of dentition images obtained using alginate impressions and IOSs were compared with the control group (plaster cast). Six different reference sites in the digital twins were compared. The mean differences in all groups were less than the 0.39 mm CBCT voxel size. Although there may be slight differences in accuracy between groups, all were clinically acceptable. The impression scan of the alginate impression two hours after impression-making (Group II) consistently displayed differences of more than 0.2 mm at all reference points, while the highest distance in the other groups was 0.053 mm. The least difference was observed when the dental image was acquired by an impression scan of an alginate impression within five minutes of impression-making (Group I) and after the segmentation scan with CS 3600 (Group III).

All digital dentition models showed inaccuracies. The plaster cast contracts and expands as the setting process progresses. The temperature and humidity of the ambient air during the setting phase affect this process. Temperatures of 20 °C and 25 °C and relative air humidity of 50 (± 10)% are recommended by the American Dental Association (ADA) and the International Organization for Standardization (ISO). Discrepancies in plaster models, ranging from 0.23 to 0.28 mm, have been extensively reported^27–30^. The dimensional stability of conventional alginate materials is also a significant concern.

Contrary to what most manufacturers advise, the alginate impressions in this study were not kept under optimal conditions of 100% humidity because scanning can be delayed in clinical settings. The dimensional stability of alginate impressions has shown errors ranging from 0.044 to 0.188 mm^31,32^. Moreover, the laser scanning process of plaster casts and alginate impressions can also induce errors^33^. The IOSs are susceptible to errors during scanning and processing. The scanning area, operator technique, expertise, and type of scanner device are all factors in the scanning errors^34^. Computer processing failures can occur as a result of flawed filter algorithms.

A limitation of this study is that the reference points used to compare the digital twins of the control and other groups were set manually. The differences in the reference points between groups may have arisen from discrepancies during the setting of the reference points. Another limitation of this study is that only participants without a history of orthodontic treatment were included because orthodontic equipment can induce artifacts and distort CBCT scans. Further, when using IOSs, the orthodontic wire and metal brackets reflect too much light and collect saliva, resulting in a longer scan time, which compromises the accuracy of the scan^34,35^. However, CBCT is often used for maxillofacial surgery or when a patient is undergoing orthodontic treatment to monitor the effectiveness of the procedure. Therefore, studies on patients using orthodontic devices are needed in the future.

## Conclusion

Compared to the plaster model, alginate impression scans and intraoral scans can supplement CBCT scans in a clinically acceptable manner. Scanning the alginate impression within five minutes of making the impression or intraoral scanning of the full arch in segments will help increase accuracy.

## Supplementary Information

Supplementary Tables.

## Data Availability

The data supporting the findings of this study are available from the corresponding authors upon reasonable request.
