# Supplementary material for: Effectiveness of creating digital twins with different digital dentition models and cone-beam computed tomography
Source: Sci Rep. 2023 Jun 30;13:10603. doi: 10.1038/s41598-023-37774-x (PMC10313775; doi:10.1038/s41598-023-37774-x)
Supplement: Supplementary file 1 — Supplementary Tables. [file 41598_2023_37774_MOESM1_ESM.docx]

Effectiveness of creating digital twins with different digital dentition models and cone-beam computed tomography

Joo-Hee Lee ^1,3,4^, Hye-Lim Lee ^1,3,4^, In-Young Park ^3,4^, Sung-Woon On ^3,4^, Soo-Hwan Byun ^2,3,4^, and Byoung-Eun Yang ^2,3,4*^

^1^ Division of Pediatric Dentistry, Hallym University Sacred Heart Hospital, Anyang 14066, Korea

^2^ Division of Oral & Maxillofacial Surgery, Hallym University Sacred Heart Hospital, Anyang 14066, Korea

^3^ Graduate School of Clinical Dentistry, Hallym University, Chuncheon 24252, Korea

^4^ Institute of Clinical Dentistry, Hallym University, Chuncheon 24252, Korea

***** Email: [face@hallym.or.kr](mailto:face@hallym.or.kr)

**Supplementary Table S1**. Statistical analysis of the coordinate value difference of each group at the lowest point of the gingival margin of maxillary right canines.

|  |  | Δx | Δy | Δz |
| --- | --- | --- | --- | --- |
| I | Average | 0.017 | 0.018 | -0.013 |
|  | SD | 0.025 | 0.032 | 0.022 |
| II | Average | 0.157 | 0.180 | 0.126 |
|  | SD | 0.041 | 0.049 | 0.038 |
| III | Average | 0.002 | 0.004 | -0.001 |
|  | SD | 0.017 | 0.022 | 0.015 |
| IV | Average | 0.009 | 0.009 | -0.002 |
|  | SD | 0.027 | 0.046 | 0.023 |
| F | | 135.720 | 98.944 | 127.782 |
| *p*(1) | | 0.000* | 0.000* | 0.000* |
| T(2) | | II > I, IV, III, C | II > I, IV, III, C | II > C, III, IV, I |

(1)statistical significances were tested by one-way ANOVA among groups (* p< 0.05).

(2)Adjustment for multiple comparisons: Tukey.

**Supplementary Table S2.** Statistical analysis of the coordinate value difference of each group at the cusp of the gingival margin of maxillary right canines.

|  |  | Δx | Δy | Δz |
| --- | --- | --- | --- | --- |
| I | Average | 0.010 | 0.036 | -0.010 |
|  | SD | 0.013 | 0.036 | 0.010 |
| II | Average | 0.162 | 0.179 | 0.129 |
|  | SD | 0.042 | 0.053 | 0.044 |
| III | Average | 0.000 | -0.002 | 0.001 |
|  | SD | 0.008 | 0.030 | 0.009 |
| IV | Average | -0.010 | -0.003 | 0.031 |
|  | SD | 0.012 | 0.025 | 0.019 |
| F | | 135.720 | 242.933 | 107.064 |
| *p*(1) | | 0.000* | 0.000* | 0.000* |
| T(2) | | II > I, C, III  C, III > IV | II > I > C, III, IV | II > IV > III, C, I |

(1)statistical significances were tested by one-way ANOVA among groups (* p< 0.05).

(2)Adjustment for multiple comparisons: Tukey.

**Supplementary Table S3.** Statistical analysis of the coordinate value difference of each group at the mesiobuccal cusp of maxillary right first molars.

|  |  | Δx | Δy | Δz |
| --- | --- | --- | --- | --- |
| I | Average | 0.006 | -0.376 | 0.000 |
|  | SD | 0.009 | 1.835 | 0.006 |
| II | Average | 0.158 | -0.195 | 0.153 |
|  | SD | 0.039 | 1.783 | 0.041 |
| III | Average | 0.006 | 0.015 | 0.000 |
|  | SD | 0.008 | 0.024 | 0.006 |
| IV | Average | -0.006 | -0.004 | 0.039 |
|  | SD | 0.017 | 0.015 | 0.016 |
| F | | 135.720 | 249.501 | 0.447 |
| *p*(1) | | 0.000* | 0.774 | 0.000* |
| T(2) | | II > III, I, C, IV |  | II > IV > III, I, C |

(1)statistical significances were tested by one-way ANOVA among groups (* p< 0.05).

(2)Adjustment for multiple comparisons: Tukey.

**Supplementary Table S4.** Statistical analysis of the coordinate value difference of each group at the lowest point of the gingival margin of maxillary left canines.

|  |  | Δx | Δy | Δz |
| --- | --- | --- | --- | --- |
| I | Average | 0.014 | -0.013 | 0.009 |
|  | SD | 0.021 | 0.018 | 0.017 |
| II | Average | 0.149 | 0.126 | 0.143 |
|  | SD | 0.046 | 0.046 | 0.047 |
| III | Average | 0.013 | -0.007 | 0.011 |
|  | SD | 0.034 | 0.037 | 0.023 |
| IV | Average | 0.000 | -0.002 | 0.005 |
|  | SD | 0.017 | 0.017 | 0.015 |
| F | | 135.720 | 102.340 | 84.449 |
| *p*(1) | | 0.000* | 0.000* | 0.000* |
| T(2) | | II > I, III, C, IV | II > C, IV, III, I | II > III, I, IV, C |

(1)statistical significances were tested by one-way ANOVA among groups (* p< 0.05).

(2)Adjustment for multiple comparisons: Tukey.

**Supplementary Table S5.** Statistical analysis of the coordinate value difference of each group at the cusp of maxillary left canines.

|  |  | Δx | Δy | Δz |
| --- | --- | --- | --- | --- |
| I | Average | -0.003 | 0.013 | -0.003 |
|  | SD | 0.010 | 0.031 | 0.007 |
| II | Average | 0.130 | 0.151 | 0.150 |
|  | SD | 0.040 | 0.046 | 0.045 |
| III | Average | 0.001 | 0.006 | -0.001 |
|  | SD | 0.005 | 0.021 | 0.008 |
| IV | Average | 0.013 | -0.022 | 0.029 |
|  | SD | 0.050 | 0.037 | 0.032 |
| F | | 135.720 | 76.490 | 98.000 |
| *p*(1) | | 0.000* | 0.000* | 0.000* |
| T(2) | | II > IV, III, C, I | II > I, III, C  C > IV | II > IV > C, III, I |

(1)statistical significances were tested by one-way ANOVA among groups (* p< 0.05).

(2)Adjustment for multiple comparisons: Tukey.

**Supplementary Table S6.** Statistical analysis of the coordinate value difference of each group at the mesiobuccal cusp of maxillary left first molars.

|  |  | Δx | Δy | Δz |
| --- | --- | --- | --- | --- |
| I | Average | 0.000 | 0.001 | 0.001 |
|  | SD | 0.007 | 0.017 | 0.004 |
| II | Average | 0.140 | 0.149 | 0.148 |
|  | SD | 0.040 | 0.051 | 0.034 |
| III | Average | -0.004 | 0.010 | -0.001 |
|  | SD | 0.006 | 0.013 | 0.003 |
| IV | Average | 0.006 | -0.008 | 0.036 |
|  | SD | 0.012 | 0.018 | 0.011 |
| F | | 135.720 | 210.946 | 133.732 |
| *p*(1) | | 0.000* | 0.000* | 0.000* |
| T(2) | | II > IV, I, C, III | II > III, I, C, IV | II > IV > I, C, III |

(1)statistical significances were tested by one-way ANOVA among groups (* p< 0.05).

(2)Adjustment for multiple comparisons: Tukey.
